# Basaloid‐Solid Adenoid Cystic Carcinoma of the Breast: A Case Report and Literature Review

**DOI:** 10.1155/crip/3701240

**Published:** 2026-03-03

**Authors:** Yijia Zhou, Yun Hu, Yansheng Zhang

**Affiliations:** ^1^ Department of Pathology, Huzhou Traditional Chinese Medicine Hospital Affiliated to Zhejiang Chinese Medical University, Huzhou, China

**Keywords:** adenoid cystic carcinoma, breast cancer, immunochemistry, pathological histology, solid-basaloid, triple-negative

## Abstract

Solid‐basaloid adenoid cystic carcinoma (SB‐AdCC) of the breast is a rare neoplasm. We report the case of a 78‐year‐old woman who presented with a progressively enlarging right breast mass over 3 years. Clinical examination revealed a 6‐cm mass with visible skin ulceration and right axillary lymphadenopathy. Imaging suggested malignancy, and biopsy showed a poorly differentiated tumor composed of basaloid cells arranged in solid nests with focal cribriform areas and necrosis. Immunohistochemically, the epithelial cells were positive for CD117 and CK7, whereas the myoepithelial component showed focal positivity for p63, CK5/6, and S100, and the tumor was negative for ER, PR, and HER2, with a Ki‐67 index of approximately 40%. These findings supported a diagnosis of SB‐AdCC of the breast with nodal metastasis. After multidisciplinary discussion, the patient underwent palliative mastectomy. At 12‐month follow‐up, local recurrence and bilateral axillary metastases were identified. This case emphasizes the diagnostic challenge of SB‐AdCC, its overlap with basaloid carcinoma, and the importance of recognizing its distinct morphological and molecular features.

## 1. Introduction

Adenoid cystic carcinoma (AdCC) is a malignant neoplasm composed of epithelial and myoepithelial cells. AdCC of the breast is extremely rare, its morphology closely resembles that of its salivary gland counterpart. Radiologic findings such as mammography and ultrasonography typically show a well‐defined or irregular mass, but imaging alone is not specific.

Solid‐basaloid adenoid cystic carcinoma (SB‐AdCC), a sparse subtype of AdCC, exhibits more aggressive characteristics, and can be challenging to diagnose [1, 2]. SB‐AdCC may mimic basaloid carcinoma or other triple‐negative breast cancers. Distinguishing those entities is critical, as they differ in prognosis and management.

Herein, we report a case of SB‐AdCC of the breast in an elderly female with a slowly enlarging mass over 3 years, share detailed clinical, histological, and immunohistochemical findings, and review the relevant literature to highlight the diagnostic considerations and biological behavior of this rare entity.

## 2. Case Report

A 78‐year‐old Asian female, with a history of hypertension and stroke, presented with a right breast lump, which was first noticed 3 years earlier. The mass had gradually increased in size, with overlying skin breakdown developing in the past year. On physical examination, a firm, immobile mass measuring approximately 6 cm was identified in the upper inner quadrant near the areola. The mass protruded from the breast surface, with associated ecchymosis and ulceration. It was easily bleeding on palpation. Nipple retraction and peau d′orange were also presented. Right axillary examination revealed enlarged lymph nodes measuring up to 5 cm in diameter, whereas the left breast and axilla were unremarkable.

Chest computed tomography (CT) demonstrated a solitary right breast mass and multiple pulmonary nodules in both lungs, the largest measuring 3.7 cm in the upper lobe of the left lung. Breast ultrasonography revealed an irregularly mixed echoic lesion, measuring 57 × 45 mm, with ill‐defined boundaries.

A vacuum‐assisted biopsy was performed on both the breast mass and the axillary lymph nodes. Pathological examination showed a poorly differentiated neoplasm with extensive necrosis. The tumor cells were arranged predominantly in a solid growth pattern with basaloid character, admixed with focal tubular and cribriform growth pattern. Glandular spaces contained bluish to pinkish myxoid material, and a myxoid stroma surround the tumor nests. Neoplastic cells were of medium‐sized, with round or oval nuclei, dense chromatin, and prominent nucleoli. Mitotic figures were approximately 3 per high‐power field (HPF). (Figure 1a,b).

Figure 1H&E stain. (a) ×200. The tumor shows solid nests with areas of necrosis and myxoid stroma. (b) ×400. The basaloid‐appearing cells show hyperchromatic nuclei and brisk mitotic activity.

**Figure:**
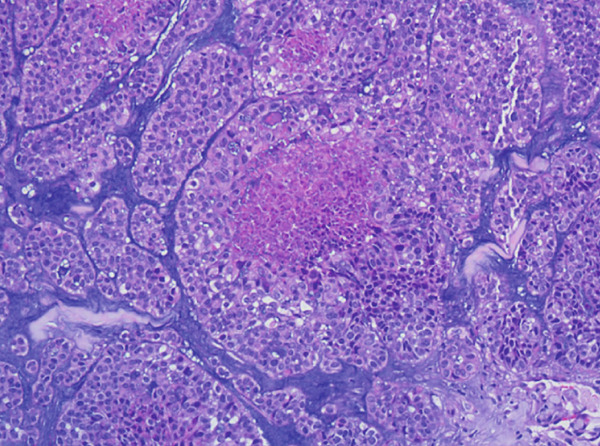
(a)

**Figure:**
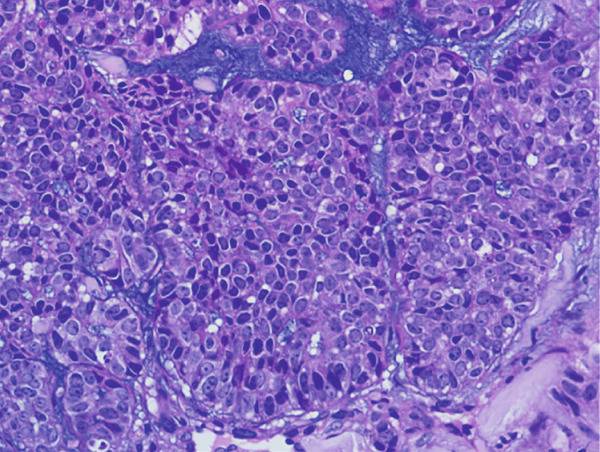
(b)

Immunohistochemical staining demonstrated a triple‐negative profile, in which estrogen receptor (ER), progesterone receptor (PR), and human epidermal growth factor receptor 2 (HER2) were negative. The tumor cells were diffusely positive for CD117 and CK7, with focal positivity for p63, CK5/6, and S100. Ki‐67 staining revealed a proliferation index of approximately 40%. The tumor was negative for CD56, Synaptophysin, and Chromogranin A. These findings supported the diagnosis of SB‐AdCC of the breast. The axillary lymph node biopsy confirmed metastatic invasive carcinoma. (Figures 2a, 2b, and 2c).

Figure 2Immunohistochemical staining, ×200. (a) CD117 shows diffuse positivity in tumor cells. (b) p63 shows focal positivity in tumor cells. (c) Ki‐67 shows approximately 40% positivity in tumor cells.

**Figure:**
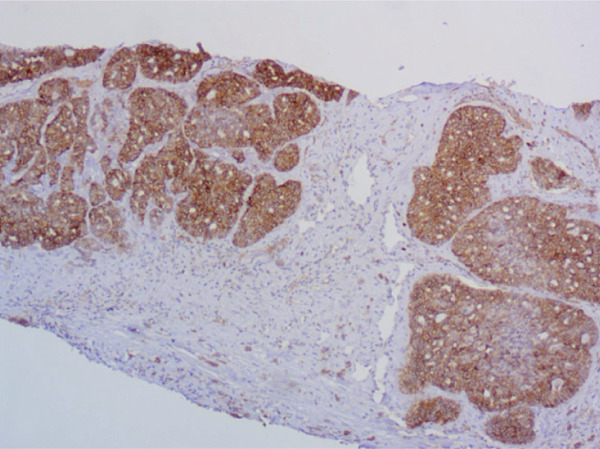
(a)

**Figure:**
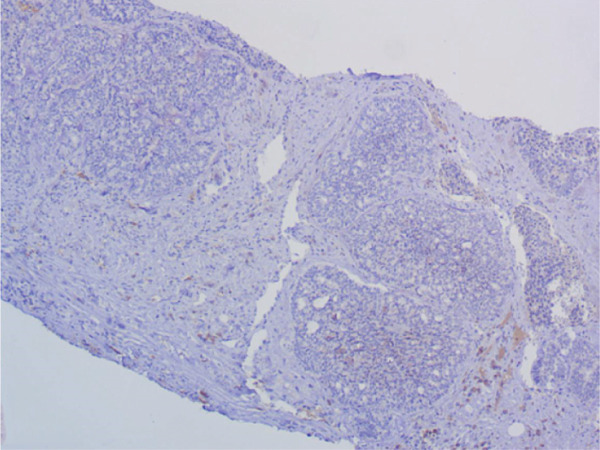
(b)

**Figure:**
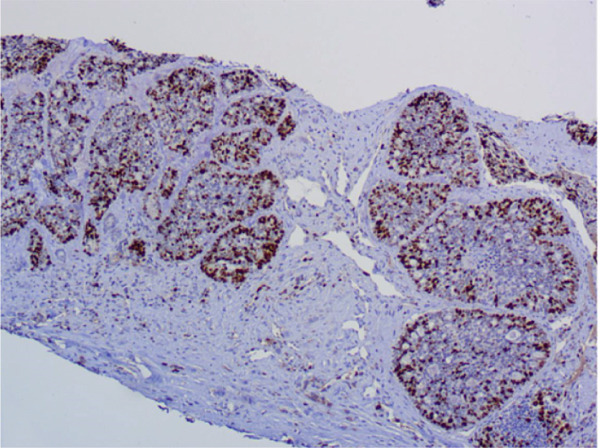
(c)

Given the extent of disease and the patient′s comorbidities, curative surgery was not feasible. After a multidisciplinary discussion and thorough communication with the patient and her family, a palliative mastectomy was performed for local disease control and symptom relief.

On gross examination, the excised specimen showed a mixed cystic‐solid lesion, with a whitish appearance, measuring 7.5 × 7 × 5.3 cm (Figure 3a,b). Histologically, the tumor exhibited infiltrative borders and demonstrated two growth patterns, approximately 60% solid and 40% cribriform, with extensive areas of necrosis. The skin and its appendages were invaded by the tumor. Perineural invasions were frequently observed (Figures 4a, 4b, and 4c).

Figure 3(a) Skin ulceration and nipple retraction are present on the tumor surface. (b) Gross specimen shows an ill‐defined cystic‐solid lesion with serous fluid in the cavity.

**Figure:**
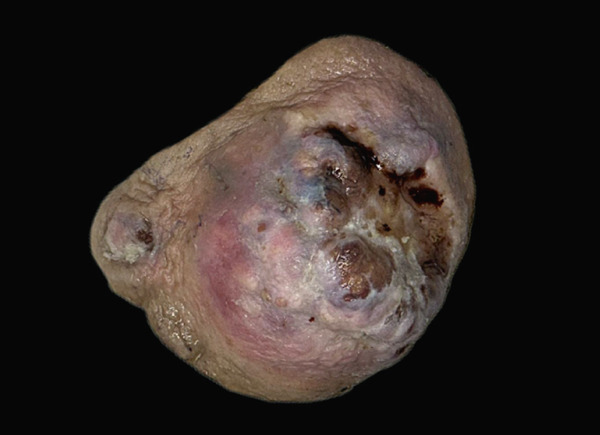
(a)

**Figure:**
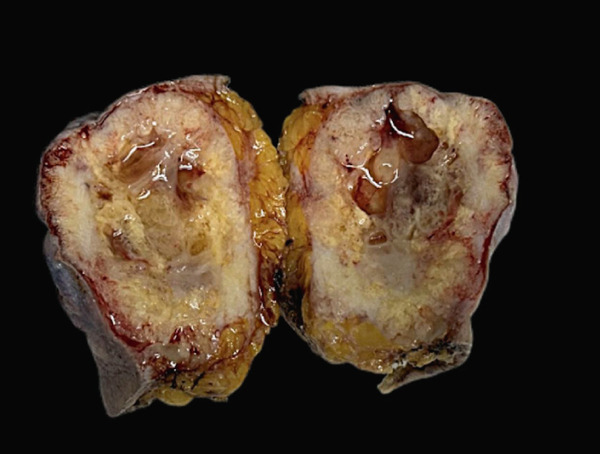
(b)

Figure 4H&E stain. (a) ×100. Two distinct growth patterns are present. (b) ×100. Epidermal invasion by tumor cells is observed. (c) ×200. Perineural invasion is present.

**Figure:**
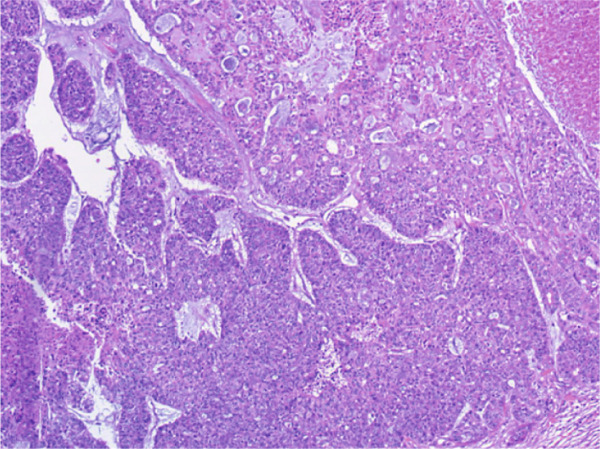
(a)

**Figure:**
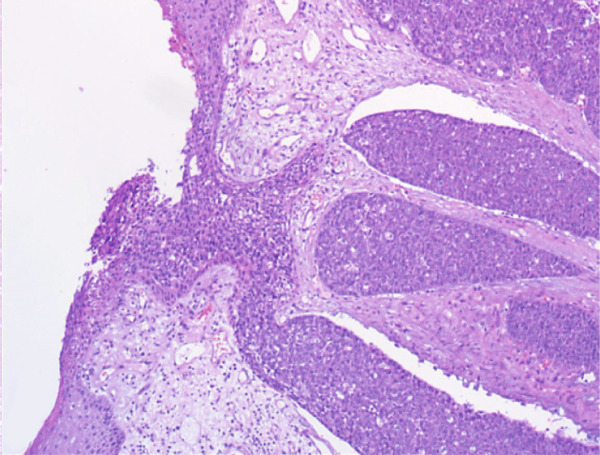
(b)

**Figure:**
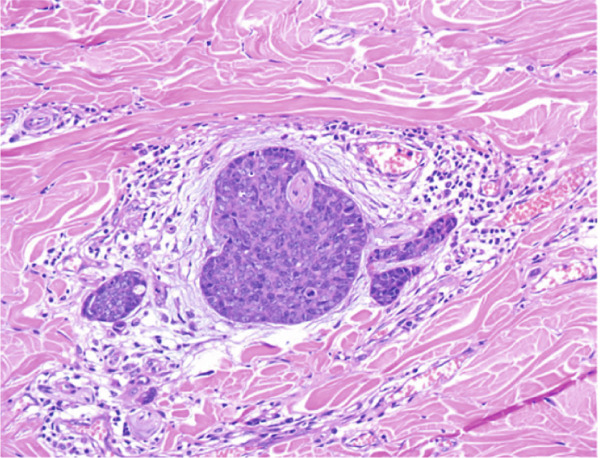
(c)

The surgical margins were negative, and axillary lymph node dissection was not performed according to the patient′s preference. The final diagnosis was SB‐AdCC of the breast. The patient refused to receive adjuvant radiotherapy and chemotherapy and remained under clinical surveillance.

At the 12‐month follow‐up, breast ultrasonography revealed local recurrence at the right chest wall and bilateral axillary lymph node metastases, indicating disease progression. The patient declined chest CT follow‐up and further systemic treatment due to personal reasons and continued under palliative care.

## 3. Discussion

AdCC of the breast is a rare neoplasm, accounting for less than 1% of all breast carcinomas. According to the World Health Organization (WHO) classification, it comprises three subtypes: conventional AdCC (C‐AdCC), SB‐AdCC, and AdCC with high‐grade transformation (AdCC‐HGT) [3]. Although C‐AdCC usually demonstrates a more favorable prognosis than other triple‐negative breast cancers, SB‐AdCC and AdCC‐HGT are considerably more aggressive, showing higher frequencies of local and distant metastases, particularly to the lungs [4, 5]. Therefore, accurate distinction among these subtypes is crucial for prognosis assessment and therapeutic decision‐making.

Histologically, breast AdCC shows a spectrum of morphologic patterns (Table 1). Although C‐AdCC, the most common subtype, generally retains well‐organized architecture and biphasic differentiation, SB‐AdCC is characterized by predominantly solid growth with higher‐grade cytologic features, which may overlap with those of other triple‐negative breast carcinomas. This morphologic overlap can pose a diagnostic challenge, particularly in limited biopsy material. AdCC‐HGT is exceedingly rare and has been well documented in the head and neck, where it is defined by the occurrence of hypodifferentiation or dedifferentiation within a pre‐existing AdCC, typically associated with loss of epithelial–myoepithelial biphasic differentiation [6–8]. In our case, the tumor displayed a solid basaloid pattern with focal cribriform areas and necrosis, findings consistent with the SB‐AdCC subtype.

**Table 1:** Histologic variants of breast adenoid cystic carcinoma.

Variant	Architectural pattern	Cytologic features	Clinical course
C‐AdCC	Predominantly cribriform, tubular and solid patterns	Low‐intermediate grade; low mitotic rate	Indolent
SB‐AdCC	Predominantly solid basaloid nests	Moderate‐marked atypia; increased mitotic rate	More aggressive
AdCC‐HGT	Abrupt high‐grade transformation	Marked atypia; high mitotic rate	Highly aggressive

In immunohistochemistry, AdCC typically exhibits a triple‐negative phenotype. However, some studies have reported occasional low‐level ER and PR expression, suggesting that it should not be routinely categorized as triple‐negative breast cancer [5, 9]. The epithelial component usually expresses CK7, CD117, and EMA, whereas the myoepithelial component shows positivity for p63, p40, calponin, SMMHC, SOX10, and S100. In our case, focal expression of p63 and S100 indicated retained myoepithelial differentiation, supporting the exclusion of high‐grade transformation. Both SB‐AdCC and AdCC‐HGT tend to exhibit higher proliferative activity than C‐AdCC.

At the molecular level, the MYB‐NFIB fusion, resulting from the t (6; 9) (q22‐23; p23‐24) translocation, represents the canonical genetic hallmark of AdCC [10]. Although MYB overexpression has also been observed in SB‐AdCC, the frequency of MYB‐NFIB rearrangement is markedly lower than that seen in C‐AdCC, suggesting alternative mechanisms of pathway activation. [2, 11] Recent sequencing studies have shown that SB‐AdCC harbors a distinct molecular profile, characterized by recurrent alterations in NOTCH1, CREBBP, and KMT2C, which are uncommon in conventional AdCC [1, 12–14], Aberrations in the NOTCH signaling pathway, together with mutations affecting chromatin‐remodeling genes, may underlie the more aggressive biological behavior of SB‐AdCC. Additionally, a novel EWSR1‐MYB fusion has recently been identified in an advanced breast AdCC exhibiting mixed classical and solid‐basaloid components. The tumor demonstrated aggressive clinical behavior, suggesting that the alternative MYB fusion may be associated with poor outcome [15]. Due to the rarity of breast AdCC‐HGT, comprehensive molecular data are currently lacking. Costa et al. described the molecular features of AdCC‐HGT in salivary glands, finding that p53 expression was significantly higher in the transformed component, suggesting potential molecular pathways involved in tumor progression [16].

In our case, distinguishing SB‐AdCC from other basaloid‐patterned breast carcinomas, including basaloid carcinoma and unclassified invasive basaloid carcinoma of the breast, was a significant diagnostic challenge, especially because the typical cribriform or tubular architecture was absent in the core biopsy. Unclassified invasive basaloid carcinoma of the breast refers to an invasive breast carcinoma showing predominant basaloid morphology that cannot be assigned to a recognized specific histologic entity. Both basaloid carcinoma and unclassified invasive basaloid carcinoma may closely mimic SB‐AdCC owing to their basaloid appearance and triple‐negative immunophenotype. However, SB‐AdCC typically demonstrates biphasic differentiation, with luminal cells, positive for CD117 and CK7, and myoepithelial cells, expressing p63 and S100, features that are usually absent in other basaloid‐patterned carcinomas. The diagnosis in our case was ultimately confirmed on the surgical specimen, which revealed classic AdCC architecture with cribriform and tubular areas embedded in a myxoid stroma. Recent studies have reported immunophenotypic and molecular overlap between SB‐AdCC and basaloid carcinoma. Although certain reports describe overlapping features between the two entitieS [13, 17], others demonstrate that MYB expression in basaloid carcinoma tends to be weaker or more focal compared with the diffuse and strong nuclear staining typically observed in SB‐AdCC [12, 18], These findings suggest that SB‐AdCC and certain basaloid carcinomas may represent entities along a biological spectrum or share related molecular pathways, although further studies are needed to clarify this relationship.

From a clinical perspective, long‐term follow‐up studies have shown that recurrence and metastasis may occur in breast adenoid cystic carcinoma, particularly in cases with positive surgical margins or more aggressive variants, such as those with high‐grade transformation. These observations highlight the prognostic relevance of histologic subtype and margin status and support careful clinicopathologic correlation in patient management [19]. This underscores the importance of long‐term follow‐up in patients with SB‐AdCC, even after initial surgical management.

## 4. Conclusion

SB‐AdCC of the breast is a rare but clinically aggressive entity. Awareness of its distinguishing features is critical to avoid misdiagnosis and to guide appropriate therapy.

## Author Contributions

**Yijia Zhou:** conception and design of the work, data collection, analysis, and interpretation, drafting the article. **Yun Hu:** data collection, analysis, and interpretation. **Yansheng Zhang:** data collection and analysis.

## Funding

No funding was received for this manuscript.

## Disclosure

Yijia Zhou and Yun Hu approved the publication of the final version.

## Ethics Statement

Ethical approval was not required because this report outlines a single case.

## Consent

Consent for publication was obtained from the patient.

## Conflicts of Interest

The authors declare no conflicts of interest.

## Data Availability

The data that support the findings of this study are available on request from the corresponding author. The data are not publicly available due to privacy or ethical restrictions.
